# MicroRNAs: emerging players in apical periodontitis

**DOI:** 10.1590/1678-7757-2020-1058

**Published:** 2021-04-14

**Authors:** Zhen SHEN, Renato Menezes SILVA

**Affiliations:** 1 University of Texas Health Science Center at Houston Department of Endodontics HoustonTX United States The University of Texas Health Science Center at Houston, Department of Endodontics, Houston, TX, United States.; 2 University of Texas Health Science Center at Houston Center for Craniofacial Research HoustonTX United States The University of Texas Health Science Center at Houston, Center for Craniofacial Research, Houston, TX, United States.

**Keywords:** MicroRNAs, Apical periodontitis, Endodontics, Biomarker

## Abstract

Apical periodontitis is an inflammatory disorder of periradicular tissues developed from endodontic infections. Understanding its pathophysiology and the underlying molecular mechanisms is key to the advancement of endodontics. MicroRNAs (miRNAs), a group of evolutionarily conserved small non-coding RNAs, may be phenotypically and functionally associated with the pathogenesis of apical periodontitis. Several studies have focused on the role of miRNAs in the pulp and periradicular biology, and they have demonstrated their essential functions, such as initiating odontogenic differentiation and promoting pro- or anti-inflammatory responses in pulpitis. Up to date, over 2,000 miRNAs have been discovered in humans; however, only few have been reported to associate with apical periodontitis. Therefore, identifying miRNAs involved in diseased apical tissues and conducting functional studies are important in expanding our current knowledge of pulp and periradicular biology and exploring novel therapeutic avenues. In this review, we revisit current models of apical periodontitis and miRNA biogenesis, analyze existing evidence of the involvement of miRNAs in diseased apical tissues, and discuss their diverse functions and potential values. Based on their sheer abundance, prolonged stability in biofluid, and relative ease of sampling, miRNAs may be a useful tool to be developed as diagnostic biomarkers for apical periodontitis. Furthermore, it can be used as therapeutic targets in conjunction with conventional endodontic therapies.

## Introduction

Microbes in the oral environment are the primary cause of infection in the root canal system as well as dental pulp and periapical tissues.^1^ Apical periodontitis – as one of the consequences of endodontic infections – is a chronic inflammatory disorder of periradicular tissues.^2^ It involves the dynamic interaction between host immune response and microbial factors at the interface between infected radicular pulp and periodontal ligament, and it is usually manifested by localized inflammation, hard tissue resorption, and destruction of other periapical tissues.^3^

Root canal therapy aims to reduce microbial infection and restore an aseptic environment to minimize apical lesions and promote bone healing. However, persistent apical periodontitis occurs when endodontic treatment has not adequately eliminated the infection or when reinfection occurs because of inadequate coronal seal or microleakage.^2,3^ Recent research advances have provided numerous molecular targets and novel endodontic techniques that may ease the development of biomarkers in early diagnosis and the translation of novel therapeutic tools to promote apical tissue regeneration. Among these targets, microRNAs – as a class of novel molecules – have recently emerged as essential regulators of these processes, and their therapeutic values have just begun to be appreciated.

### Pathophysiology of apical periodontitis

The exact etiology of apical periodontitis has not been well elucidated. Evidence has shown that bacteria – especially Gram-negative bacteria – and their toxins, enzymes, and metabolic byproducts play a significant role in inducing host immune response.^4^ Lipopolysaccharides (LPS) or endotoxin, is a major component of the cell wall of Gram-negative bacteria.^4^ It is often secreted by growing organisms or released during the disintegration of bacteria.^4^ It is one of the most important virulence factors and potent stimuli of immune cells such as macrophages, with the lipid A moiety responsible for the toxic effects of LPS.^5^ LPS evokes host immune response by activating pattern recognition receptors (PRRs) such as Toll-like receptors (TLRs) and CD14 signaling. These signaling events subsequently induce the production of pro-inflammatory cytokines, such as tumor necrosis factor-alpha (TNF-α), interleukin-1 (IL-1), IL-8, IL-12, IL-17, and anti-inflammatory cytokines such as IL-4 and IL-10.^5-7^

Periapical inflammatory responses can cause tissue damage and the development of granulomas or radicular cysts by a complex cellular and molecular cascade.^8^ For instance, matrix metalloproteinases (MMPs) – a group of more than 25 secreted and membrane-bound enzymes – are regulated by pro-inflammatory cytokines and growth factors, and play one of the key roles in tissue destruction and normal tissue modeling.^9^ In particular, MMP-1, MMP-2, MMP-3, MMP-8, MMP-9, and MMP-13 have been found in periapical lesions. Collagen, as well as extracellular matrix (ECM) degradation, have been associated with increased levels of MMPs.^9^

In addition to pathophysiological changes in connective tissues, periapical inflammation also leads to alveolar bone resorption.^3^ Bone turnover depends on the equilibrium between osteoblast-mediated bone formation and osteoclast-mediated bone destruction, and the osteoclast activities are regulated by the receptor activator of NF-kappa B (RANK), its ligand RANKL, and the decoy receptor of RANKL, osteoprotegerin (OPG).^10^ The binding of RANKL to RANK drives the maturation of osteoclasts. Studies have shown that pro-inflammatory cytokines can induce the expression of RANKL, which leads to increased osteoclast activities and subsequent bone resorption.^10^

It is noteworthy that complex interactions between inflammatory cells and cytokines influence the balance between pro- and anti-inflammatory processes that, in turn, affect the equilibrium between destruction and regeneration of apical tissues.^11^ For example, anti-inflammatory cytokines, such as IL-4 and IL-10, can restrain inflammatory cytokine production and inhibit the function of MMPs and RANKL by stimulating the production of tissue inhibitors of matrix metalloproteinases (TIMPs) and OPG, respectively.^12^

### MicroRNA

MicroRNAs (miRNAs) are a class of evolutionally conserved non-coding RNAs of ~23 nucleotides that regulate gene expression predominantly at the post-transcriptional level.^13^ Since its initial discovery of lin-4 in *Caenorhabditis elegans* in 1993, hundreds of miRNAs have been identified and extensively studied in a wide spectrum of species.^14,15^

Mammalian miRNAs are encoded in both intergenic and intragenic regions of the genome.^16^ In general, miRNA genes are first transcribed into long primary transcripts, called pri-miRNAs. Pri-miRNAs are processed by the nuclear RNase III Drosha/DGCR8 to generate ∼70 base pair-long pre-miRNAs that are then exported by Exportin-5 to the cytoplasm in a Ran-GTP dependent manner.^17^ In the cytoplasm, pre-miRNAs are further processed by the RNase III Dicer to yield ~21-25 nucleotide-long mature miRNAs.^17^ They bind to the 3’ untranslated region (UTR) of the target messenger RNAs (mRNAs) via partial sequence complementarity and subsequently lead to translational inhibition and mRNA decay of the target genes.^13^

MiRNAs play key roles in development, as evident from early mouse model studies that knockout of miRNA processing proteins causes embryonic lethality.^18^ Over the past two decades, miRNAs have also been associated with many dental pulp disorders and diseases, with many studies shedding light on their potential diagnostic and prognostic values.^19^

### MicroRNAs in apical periodontitis

Recent miRNA profiling studies have shown that miRNAs play an important role in the pathogenesis of endodontic apical periodontitis.^20-22^ Gao and Zheng^20^ (2013), investigated the differential expression of miRNAs in rat apical periodontitis via microarray analysis, and target genes of these miRNAs and their biological functions were predicted. Interestingly, particular enrichment in the MAPK signaling pathways was reported. Chan, et al.^21^(2013) identified 381 miRNAs from diseased periapical tissues collected from patients undergoing endodontic surgery compared with those of healthy pulps using microarray analyses, and they found that 24 miRNAs were downregulated in diseased periapical tissues compared with controls. They further confirmed seven downregulated miRNAs using quantitative real-time polymerase chain reaction (PCR) and predicted their targets that include key mediators in the immune and inflammatory response, such as IL-6, MMP-9, and TGF-β.^21^

Although a large number of miRNAs have been identified in apical periodontitis, only few were further studied and characterized. For example, Yue, et al.^22^ (2016) found that miR-155 played a key role in inflammation and the progression of apical periodontitis by inhibiting SEMA3A. In another study, Yue, et al.^23^ (2017) revealed that miR-335-5p acted as a positive mediator in human periodontal ligament fibroblasts (HPDLFs) inflammation and identified two targets, uPAR and RANKL. They found that uPAR was repressed by miR-335-5p at the basal level, but when HPDLFs were subjected to LPS stimulation, it could be relieved from miR-335-5p-mediated repression, a process called derepression; whereas miR-335-5p promoted RANKL in HPDLFs regardless of whether or not it was under inflammatory conditions.^23^

Lina, et al.^24^ (2019) discovered miR-146a and Hey2 (hairy and enhancer-of-split related with YRPW motif 2) expressions were significantly higher in 20 patients with chronic apical periodontitis. Moreover, they elucidated miR-146a acted as an anti-inflammatory mediator and negatively regulated the expression of IL-6, IL-1β, and TNF-α.^24^ They also confirmed Hey2 was a target gene of miR-146a and negatively regulated miR-146a, IL-6, IL-1β, and TNF-α expressions, suggesting that miR-146a and Hey2 form a mutual negative feedback regulatory loop in chronic apical periodontitis.^24^

Wang, et al.^25^ (2019) reported that miR-181b-5p was downregulated in TNF-α-stimulated cementoblasts, and overexpressing miR-181b-5p negatively regulated IL-6 and pro-inflammatory chemokine, indicating miR-181b-5p moderates pro-inflammatory chemokine production by targeting IL-6 in cementoblasts. Interestingly, they found miR-181b-5p promoted cementoblast apoptosis, which may enhance the resolution of inflammation.^25^

Recently, the association between polymorphisms in genes encoding estrogen receptors 1 and 2 (ESR1 and ESR2), vitamin D receptor (VDR), and in miRNA-17 with apical periodontitis was reported in the literature. This study included 162 patients who had received endodontic therapy. Among them, 89 patients were considered “healed,” whereas 73 patients were diagnosed with persistent apical periodontitis at the follow-up visit. They found that genetic polymorphisms in ESR1 (rs2234693 and rs9340799), ESR2 (rs1256049 and rs4986938), VDR (rs739837 and rs2228570), and miRNA-17 (rs4284505) were not associated with persistent apical periodontitis.^26^

Not only did these findings confirm the diverse and complex miRNA regulatory network in apical periodontitis, but they also indicated that a few potential areas of study may be conducted in the future to further advance our understanding of miRNAs.

First, differential expression patterns of miRNAs may be cell type-specific. As mentioned before, apical periodontitis involves several cell types, such as polymorphonuclear leukocytes, macrophages, lymphocytes, plasma cells, fibroblasts, epithelial cells, and vascular cells.^27^ As a result, miRNA profiling may reflect a pool of different patterns. It is highly possible that some miRNAs are only expressed or differentially expressed in certain cell types. Therefore, cell type-specific miRNA profiling will have great significance. An excellent example comes from Naqvi, et al.^28^ (2016) that performed miRNA profiling in primary human macrophages challenged with LPS, and they found that these miRNA profiles were responsive to LPS dose and incubation time.^28^ Recent advancement in the single-cell RNA sequencing technology is also likely to generate significant insights into the cellular heterogeneity of apical periodontitis and in-depth molecular signatures.

Second, the development of sequencing techniques – the next-generation miRNA sequencing (miRNA-seq) – will significantly change our understanding of miRNAs. Compared with miRNA profiling studies using microarray analysis, miRNA-seq eliminates the requirement of sequence-specific probes and enables an unbiased study of miRNA expression landscape and discovery of novel miRNAs.^29^ Recently, our group performed a large-scale next-generation miRNA-seq analysis of human diseased and healthy apical tissues to identify differentially expressed miRNAs in apical periodontitis.^30^ As a result, a total of 852 miRNAs were identified, out of which 12 were significantly upregulated (1.54-8.44 fold), and 94 were significantly downregulated (0.14-0.67 fold). Moreover, 515 novel miRNAs expressed an appreciable level.^30^

Third, once the differentially expressed and/or novel miRNAs are identified, functional studies will become the key in determining how these miRNAs contribute to the pathophysiology of apical periodontitis. Immunohistochemistry and fluorescence *in situ* hybridization may give us clues on the specific cell types in which those miRNAs reside. In addition, miRNA mimics and inhibitors can be readily synthesized and introduced into *in vitro* and *in vivo* experimental systems; changes of predicted miRNA target genes can be examined at the mRNA levels and protein levels via molecular biology and biochemistry techniques such as real-time PCR, western blotting, and enzyme-linked immunosorbent assay (ELISA); and biological pathways involving those target genes can then be established. Furthermore, using *in vitro* luciferase assays or similar techniques, direct target genes of differentially expressed miRNAs can be easily screened out. For example, our recent miRNA-seq analysis revealed that miR-10a-5p was highly expressed in apical periodontitis, and overexpression of miR-10a-5p *in vitro* resulted in the downregulation of TNF-α and upregulation of IL-10, indicating that this most significantly upregulated miRNA in apical periodontitis may function in suppressing inflammation and promoting healing.^30^ Therefore, functional characterization of miRNAs plays a pivotal role in understanding the pathophysiology and will ease the development of biomarkers and therapeutic targets in apical periodontitis.

### MicroRNAs as biomarkers for apical periodontitis

MiRNAs have been used as biomarkers in many diseases and medical conditions to aid diagnosis and prognosis. The same concept can be applied to miRNAs in apical periodontitis as chairside diagnostic tools based on their sheer abundance, prolonged stability, and relative ease of sampling.^31^

**Figure 1:**
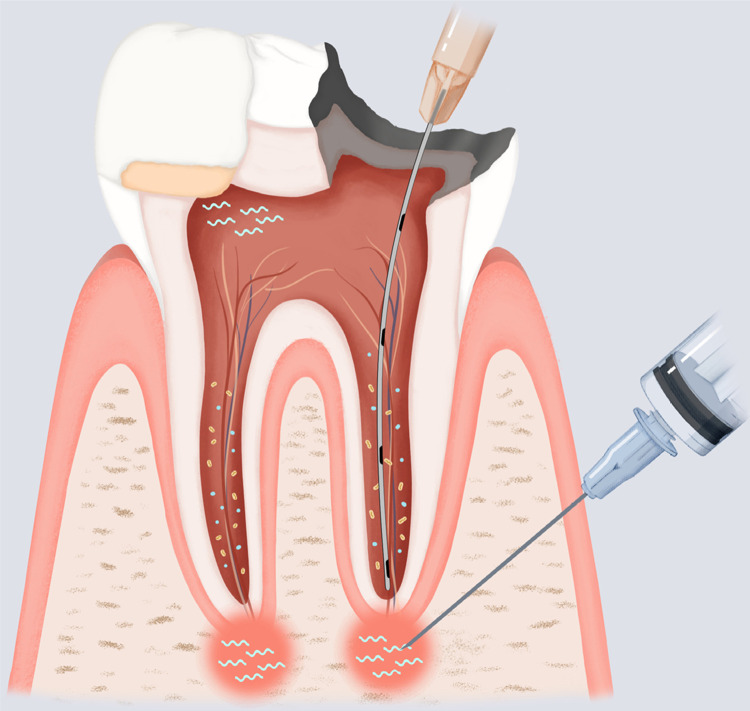
Potential clinical translation for the use of miRNAs in the treatment of pulpal and periapical diseases.

For example, when pulp exposure is encountered during caries excavation, pulpal fluid or blood can be easily sampled using instruments such as syringes or microcannulas, and miRNA levels can be assessed. If specific pro-inflammatory miRNAs are detected at increased levels, this may indicate a substantial extent of inflammation. Therefore, direct pulp capping or other vital pulp therapies may have a poor prognosis in this situation, and non-surgical root canal therapy may be the recommended treatment option. Conversely, the detection of high levels of anti-inflammatory miRNAs may indicate a good prognosis of performing vital pulp therapy (Figure 1).

As many novel miRNAs are being unveiled, they have the potential to be translated into chairside diagnostic biomarkers and therapeutic agents. For example, before any endodontic treatment, pulpal/periapical fluid and blood can be easily sampled using instruments such as syringes or microcannulas, from which miRNA levels can be assessed. Based on the differential expression patterns of specific miRNAs that are associated with characteristics and/or prognostic factors of apical periodontitis, informed decision-making can be achieved, and patient-centered treatment options can be tailored and rendered. On the other hand, if specific miRNAs are known to play a role in pro- and anti-inflammatory process, their inhibitors/mimics can be applied to inflamed pulp tissues in conjunction with conventional vital pulp therapies such as direct pulp capping or delivered to the root canals and periapical regions using microcannulas during root canal therapies to promote better healing outcomes.

This concept can also be used for non-surgical root canal treatment. Similarly, pulpal and periapical fluid can be obtained during the procedure. If high levels of certain pro-inflammatory miRNAs are detected in the apical area, it may shift the prognosis of healing to questionable. In this case, a surgical approach following non-surgical root canal treatment can be discussed in advance and planned if necessary (Figure 1). This will greatly enhance the informed decision-making process, and patient-centered treatment options can also be better tailored and rendered.

In addition, if miRNA profiling can be further stratified based on symptomatology (symptomatic vs. asymptomatic) or severity of the symptoms, this would aid our prediction of pre-/post-op pain levels and better facilitate pain management for patients.

### MicroRNAs as therapeutic targets for apical periodontitis

Similar to the idea of biomarkers, miRNAs can also be designed as therapeutic targets.^31^ As hinted at above, inhibitors of pro-inflammatory miRNAs or mimics of anti-inflammatory miRNAs can be used in conjunction with traditional endodontic therapies. This type of treatment aims to prevent or inhibit the inflammatory process in apical periodontitis (Figure 1). It was recently demonstrated that miR-10a-5p may suppress inflammation in apical periodontitis.^30^ If this can be further shown with *in vivo* studies, then miR-10a-5p has the potential to be developed as a therapeutic agent and be used as an adjunct therapy along with the conventional endodontic treatment.

Currently, several miRNA-based therapeutics are undergoing clinical trials targeting diseases such as hepatitis C, scleroderma, mesothelioma, non-small cell lung cancer, cutaneous T cell lymphoma, and mycosis fungoides.^32^ These all shed light on the potential use of miRNAs in treating apical periodontitis. One of the major challenges is the degradation of exogenous miRNA products (miRNA mimics and inhibitors) by endogenous nucleases. Chemical modifications, such as the addition of a 2’-O-methyl group or locked nucleic acids (LNAs), may significantly increase their stability. Another challenge is the targeted delivery of miRNA products to the disease sites. Lipid nanoparticles is one of the commonly used systems for the delivery of small molecules and have been developed for delivering miRNAs to target sites.^32^ Compared to cancers and some other diseases, apical periodontitis typically presents localized diseased tissues, which makes it easier for clinicians to target it. Thus, miRNA products may be distributed via intracanal delivery using a microcannula or by local infiltration using a syringe during non-surgical root canal treatment (Figure 1), or by direct application during apical surgery. Despite all the technical hurdles, the identification and functional study of miRNAs is significant.

What if the damage already exists, such as bone resorption and diseased tissue formation? Can miRNAs help repair or regenerate destructed tissues? Studies on the functions of miRNAs in the odontogenic differentiation of stem cells may give us a clue.^19,33^ Taking human dental pulp stem cells (DPSCs) as an example, several miRNAs are associated with their odontogenic potentials. For instance, miR-21 and STAT3 (signal transducer and activator of transcription 3) formed a positive reciprocal feedback loop in the signaling pathway that enhanced the process of odontogenic differentiation of DPSCs.^34^ MiR-223-3p was significantly upregulated in inflamed dental pulp, and the overexpression of miR-223-3p in DPSCs significantly increased the protein levels of dentine sialophosphoprotein (DSPP) and dentin matrix protein 1 (DMP-1), thus promoting odontoblast differentiation.^35^ In contrast, some miRNAs show anti-odontogenic differentiation properties. Downregulation of miR-143-5p promoted the differentiation of DPSCs into odontoblasts by enhancing Runx2 expression via the OPG/RANKL signaling pathway and by activating the p38 MAPK signaling pathway.^36,37^ Similarly, the downregulation of miRNA-488 also enhanced the odontoblast differentiation of DPSCs via activation of the p38 MAPK signaling pathway.^38^ Another miRNA, miR-140-5p was significantly decreased during LPS-mediated differentiation of DPSCs *in vitro*, and overexpression of miR-140-5p enhanced DPSC proliferation and inhibited DPSC differentiation.^39^

As many known miRNAs being characterized and novel miRNAs being unveiled in apical periodontitis, it is promising that more therapeutic targets within this family of small non-coding RNAs will be identified, and apical periodontitis will be tackled from multiple biological pathways such as suppression of inflammation, promotion of wound healing, and differentiation of stem cells. A MiRNA cocktail therapy may not be an unrealistic assumption.

## Conclusion

Understanding the biology of pulpal and periradicular tissues is key to the advancement of endodontics. The central dogma of DNA-RNA-Protein axis has been well studied over the past half-century. However, miRNAs, as a class of novel non-protein coding RNAs, have just begun to be appreciated. Compared to other cellular processes, fewer studies have focused on the role of miRNAs in pulp and periradicular biology but have already demonstrated their essential functions. Therefore, identifying miRNAs in apical periodontitis and conducting functional studies are important in unveiling the big picture and expanding the current knowledge of pulp biology. Furthermore, based on the sheer abundance, prolonged stability, and relative ease of sampling, miRNAs may be a useful tool to be developed as diagnostic biomarkers for apical periodontitis and can be used as biological materials in conjunction with conventional endodontic therapies.
